# Annual Medication Use and Costs Among Children

**DOI:** 10.1001/jamanetworkopen.2025.1529

**Published:** 2025-03-24

**Authors:** Anowara Begum, Patrick Hosokawa, Lucas E. Orth, James A. Feinstein

**Affiliations:** 1 Adult and Child Center for Outcomes Research and Delivery Science, University of Colorado and Children’s Hospital Colorado, Aurora; 2 Skaggs School of Pharmacy and Pharmaceutical Sciences, University of Colorado, Aurora; 3 Department of Pediatrics, University of Colorado, Aurora

## Abstract

This cross-sectional study analyzed outpatient prescription medication use and cost among children in Colorado and identified those who would qualify for medication therapy management under Centers for Medicare and Medicaid guidelines.

## Introduction

Children with chronic medical conditions often take multiple medications, which can lead to medication-related problems.^1^ For adults, medication therapy management (MTM) programs are provided free to Medicare beneficiaries who exceed an annual prescription cost threshold. MTM consists of a pharmacist-led comprehensive medication review, medication optimizations, and creation of a medication action plan.^2^ MTM has improved patient safety and reduced medication spending, but no similar MTM interventions exist for children.^2^ We aimed to (1) describe annual outpatient prescription medication use and costs among children and (2) determine which children would qualify for MTM based on the established Centers for Medicare and Medicaid Services (CMS) threshold.^2^

## Methods

We conducted a cross-sectional study of youths aged 1 to 21 years who were prescribed 1 or more medications in the 2022 Colorado All Payer Claims Database. Polypharmacy level was defined by the maximum count of concurrently used medications (no polypharmacy, 1 medication; low, 2-4; high, ≥5).^3^ Annual total medication costs per patient were calculated using the pharmacy’s price from a manufacturer.^4^ Medication classes were assigned using the Anatomical Therapeutic Classification system. Medical complexity was assessed using the Complex Chronic Condition (CCC) system.^5^ Multivariable logistic regression was used to test for patient characteristics (age, CCCs, and health care utilization) associated with exceeding the 2022 CMS MTM cost threshold (≥$4622 annually).^2^ Analyses were conducted using Stata, version 18.5 (StataCorp LLC) and *P* < .05 was considered significant. Colorado Multiple Institutional Review Board approved this study with a waiver of informed consent because deidentified data were used. We followed the STROBE reporting guideline.

## Results

Of 529 055 children prescribed 1 or more medications (mean [SD] age, 10.9 [5.9] years; 51% [269 454] were female), 45% (235 308) were aged 13 to 21 years, 50% (262 911) had no polypharmacy, 44% (234 842) had low polypharmacy, and 6% (31 302) had high polypharmacy (Table). Compared with those with no polypharmacy, patients with high polypharmacy were more likely to be female (57% vs 50%), to be aged 13 to 21 years (73% vs 34%), and to have any CCC (37% vs 11%) (all *P* < .001). They also had more inpatient (8% vs 1%), emergency (35% vs 23%), and outpatient visits (54% vs 34%) (all *P* < .001).

**Table.** Patient Demographics, Clinical Characteristics, and Medication Utilization by Level of Polypharmacy in the Colorado All Payer Claims Database, 2022

	Total No. (%) (N = 529 055)	No. (%) by level of polypharmacy^a^		
		No (n = 262 911)	Low (n = 234 842)	High (n = 31 302)
Patient demographic				
Age, y				
1	27 669 (5.2)	17 316 (6.6)	9876 (4.2)	477 (1.5)
2-8	173 506 (32.8)	105 334 (40.1)	64 065 (27.3)	4107 (13.1)
9-12	92 572 (17.5)	51 600 (19.6)	37 000 (15.8)	3972 (12.7)
13-21	235 308 (44.5)	88 661 (33.7)	123 901 (52.8)	22 746 (72.7)
Sex				
Male	259 601 (49.1)	132 280 (50.3)	113 735 (48.4)	13 585 (43.4)
Female	269 454 (50.9)	130 631 (49.7)	121 107 (51.6)	17 717 (56.6)
Clinical characteristic				
Any CCC	80 195 (15.2)	28 915 (11.0)	40 582 (17.3)	11 418 (36.5)
CCC count				
0	448 223 (84.7)	234 028 (89.0)	194 301 (82.7)	19 894 (63.6)
1	58 366 (11.0)	23 399 (8.9)	29 403 (12.5)	5664 (18.1)
2	13 430 (2.5)	3895 (1.5)	7067 (3.0)	2468 (7.9)
≥3	9036 (1.7)	1689 (0.6)	4071 (1.7)	3276 (10.5)
Visit count				
0	326 183 (61.7)	174 467 (66.4)	137 297 (58.5)	14 419 (46.1)
1	61 677 (11.7)	31 026 (11.8)	27 423 (11.7)	3228 (10.3)
2-4	81 987 (15.5)	37 712 (14.3)	38 901 (16.6)	5374 (17.2)
5-9	37 259 (7.1)	13 769 (5.2)	20 025 (8.5)	3735 (11.9)
≥10	21 679 (4.1)	5937 (2.3)	11 196 (4.8)	4546 (14.5)
Emergency visit count				
0	393 095 (74.3)	203 078 (77.2)	169 702 (72.3)	20 315 (64.9)
1	82 418 (15.5)	38 974 (14.8)	38 051 (16.2)	5393 (17.2)
2	30 530 (5.8)	13 062 (5.0)	14 928 (6.4)	2540 (8.1)
≥3	23 012 (4.4)	7797 (3.0)	12 161 (5.2)	3054 (9.8)
Inpatient visit count				
0	516 688 (97.7)	259 410 (98.7)	228 506 (97.3)	29 772 (91.9)
1	10 599 (2.0)	3264 (1.2)	5565 (2.4)	1770 (5.7)
2	1768 (0.3)	237 (0.1)	771 (0.3)	760 (2.4)
Medication utilization				
Total annual medication costs, $	1 111 317 842	108 898 265	480 566 431	521 853 146
Total annual medication costs, median (IQR)	83 (16-430)	25 (8-100)	233 (51-846)	2876 (658-8754)
Total annual prescription counts, median (IQR)	3 (1-6)	1 (1-2)	5 (3-9)	25 (14-40)

Abbreviation: CCC, complex chronic condition.

^a^ Column percentages may not sum to 100 due to rounding.

^b^ Test of comparisons were all significant (*P* < .001) across levels of polypharmacy for each row variable.

Of $1.1 billion spent on medications, 47% ($521 853 146) of expenditures were attributed to patients with high polypharmacy. The costliest medication classes (Figure) were neurological ($242 million), respiratory ($142 million), and gastrointestinal ($112 million). Patients with no polypharmacy filled a median (IQR) of 1 (1-2) prescription annually, with a median (IQR) annual medication cost of $25 ($8-$100). Those with high polypharmacy filled a median (IQR) of 25 (14-40) prescriptions, with a median (IQR) annual cost of $2876 ($658-$8754). Five percent (26 386) of patients exceeded the cost threshold for MTM; associated factors were older age (13-21 years vs 2-8 years; OR, 3.8; 95% CI, 3.6-3.9), having more CCCs (≥3 vs 0; OR, 11.4; 95% CI, 10.8-12.1), higher inpatient visit counts (≥2 vs 0; OR, 2.6; 95% CI, 2.3-2.9), and higher outpatient visit counts (≥10 vs 0; OR, 2.4; 95% CI, 2.3-2.5).

**Figure.**
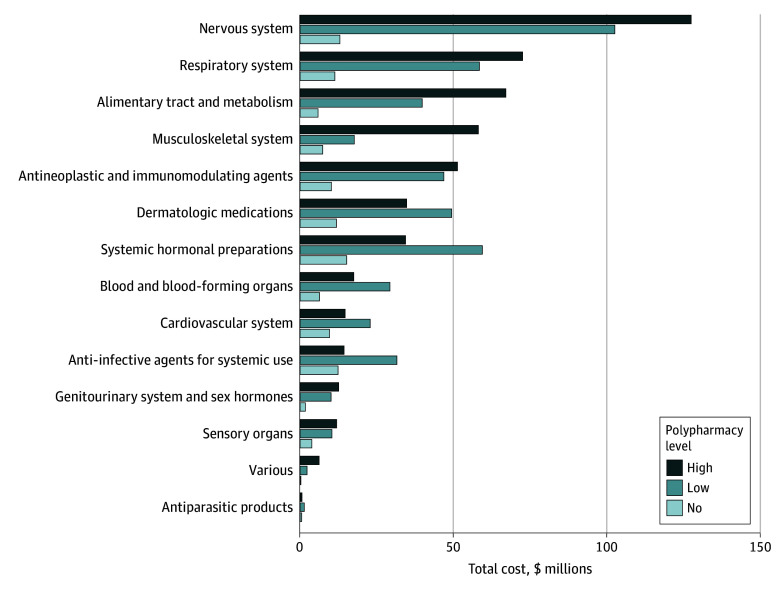
Medication Classes by Cost and Level of Polypharmacy in the Colorado All Payer Claims Database in 2022 Total expenditures for each medication class in the Anatomic Therapeutic Classification system.

## Discussion

Five percent of Colorado children prescribed 1 or more medications exceeded the CMS cost threshold for MTM, most frequently for adolescents with high polypharmacy and medical complexity. Study limitations included that we could not account for dispensing fees and sales tax or identify as-needed medications. Nevertheless, these findings are consistent with prior research describing that children with polypharmacy have myriad opportunities to reduce medication-related problems.^6^ However, current management of pediatric polypharmacy is ad hoc, reactive, and problem-focused rather than proactive, structured, and comprehensive.^3^ Among children who reach the adult CMS cost threshold, MTM has potential utility, and this need could grow based on a reduced CMS cost threshold of $1623 in 2025.^2^ Pediatric health care systems and payers should prioritize pharmacist-led pediatric MTM for the highest-need patients, possibly targeted by diagnosis. Adapting adult models for shared or virtual MTM services could improve effectiveness, safety, and cost of medication regimens for children and youth.

## Notes

Supplement.
Data Sharing Statement
