# Supplementary material for: Annual Medication Use and Costs Among Children
Source: JAMA Netw Open. 2025 Mar 24;8(3):e251529. doi: 10.1001/jamanetworkopen.2025.1529 (PMC11933993; doi:10.1001/jamanetworkopen.2025.1529)
Supplement: Supplement. — Data Sharing Statement [file jamanetwopen-e251529-s001.pdf]

Data Sharing Statement

Begum. Annual Medication Use and Costs Among Children. *JAMA Netw Open*. Published March 24, 2025. doi:10.1001/jamanetworkopen.2025.1529

Data
Data available: No
